# Emergency Physician Assessment of Productivity and Supervision Practices

**DOI:** 10.5811/westjem.19417

**Published:** 2025-04-01

**Authors:** Kraftin Schreyer, Diane Kuhn, Vicki Norton

**Affiliations:** * Lewis Katz School of Medicine at Temple University, Department of Emergency Medicine, Philadelphia, Pennsylvania; † Indiana University Health, Department of Emergency Medicine, Indianapolis, Indiana; ‡ Florida Atlantic University, Department of Emergency Medicine, Boca Raton, Florida

## Abstract

**Introduction:**

Despite a lack of data guiding safe standards for physician productivity and supervision of non-physician practitioners (NPP), legislation dictating supervision ratios for emergency physicians (EP) has been enacted in Florida and elsewhere across the country. To inform future legislation, we aim to identify current productivity and supervision practices among practicing EPs as well as those physicians’ safety assessments of their current practices.

**Methods:**

We conducted a cross-sectional observational study regarding EPs’ perspectives on safe staffing and supervision models. A survey, consisting of 14 questions examining different variables affecting supervision and productivity, was used to determine physicians’ opinions on the safety of productivity and supervision models across a range of annual volumes, employers, and years of experience. We coded safety assessments as binary (yes/no) and measured productivity by patients treated per hour. Ratios of physician to supervisee (either resident physician or or NPP) were given as number of supervisees: EP.

**Results:**

The survey response rate was 4.8% (196/4,004). On average, most EPs treated 2.6 patients per hour, regardless of years of experience, employment model, or supervision model. More than 80% of EPs felt that their current patients-per- hour practice was safe. Direct supervision represented 59% of total visits and the majority in all employment models except for community contract-management groups (CMG). A minimum of 80% of physicians felt that their current supervision practices were safe across employment models, with the notable exception of community CMGs. Most felt that a safe ratio for direct supervision of NPPs was 1:1. Over 30% reported there was no safe staffing ratio for indirect supervision.

**Conclusion:**

With the exception of those employed by community contract-management groups, EPs felt that their current productivity and supervision practices were safe; however, average productivity and supervision ratios are much lower than prior estimates and in current legislation governing emergency department practice. Standards of care for both productivity and supervision that take into account current practices and safety assessments should be established and considered when future policies and legislation are developed.

## INTRODUCTION

### Background

Over the past several years, state legislatures have considered numerous bills related to physician scope of practice and supervision, with one common area of legislation related to the number of nurse practitioners (NP) or physician assistants (PA) that a physician may supervise simultaneously.^1^,^2^ For example, in 2021 Florida increased the number of physician assistants a doctor may supervise from 4 to 10, with no stated limit on the number of NPs.^3^,^4^ Despite the frequency of introduction and importance of these policies to patient care, little is known about safe practices regarding physician supervision of NPs and PAs (collectively non-physician practitioners, or NPPs).^5^,^6^ Furthermore, there is very limited recent data on physician productivity levels more generally, particularly in an emergency department (ED) setting where care occurs in an unscheduled manner.^7^

Some previous work has been limited by the operationalization of patient safety with “rare and proxy outcomes that probably represent only the ‘tip of the iceberg.’”^5^,^8^ Thus, there is a significant gap in the literature regarding current emergency physician (EP) productivity and supervision practices, as well as safety assessments of these practices. Ideally, safety of productivity and supervision models might be measured in terms of patient outcomes, although the challenges of such an approach have been well documented.^9^,^10^ Thus, EPs’ own assessments of the safety of their supervision and productivity can contribute to overall understanding.

### Objectives

Given the evolution of emergency medicine (EM) and recent legislation impacting emergency care, there is a need for an assessment of current practice patterns. Our primary objective in this study was to determine the current productivity and supervision practices of practicing EPs. Secondarily, we aimed to assess the perceptions of those practices by the same EPs.

## METHODS

### Study Design and Setting

This was a cross-sectional observational study regarding EPs’ perspectives on safe staffing and supervision models conducted from November–December 2023. This study was conducted on behalf of the American Academy of Emergency Medicine (AAEM). The survey was designed by a task force of the AAEM Workforce and Operations Management committees, which developed the questions and responses and incorporated committee revisions prior to distribution. It consisted of 14 questions examining different variables affecting supervision and productivity. The survey (Appendix A) was distributed electronically to the AAEM listserv, which includes 4,044 EPs. One additional reminder email was sent to the listserv to encourage participation in the survey.

### Variables and Measures

Independent variables included the following: 1) physician sex; 2) physician race; 3) physician ethnicity; 4) physician years out of residency; 5) employment model; 6) practice setting; 7) annual volume at primary practice setting (<20,000 patients annually, 20,001–50,000, 50,001–75,000, 75,001–100,000, more than 100,000); and 8) number of patients seen per hour at primary workplace under a direct, indirect, and unsupervised/retrospective chart review model. Physician years out of residency data was collected as a ratio variable and recoded as categorical for analyses. Listed employment models included the following: contract management group (CMG), for which Envision, TeamHealth, and SCP were listed as prototypes; democratic group; hospital employed; or military. Practice settings were community and academic.

**Population Health Research Capsule**

What do we already know about this issue?

*Legislation on physician supervision of non-physician practitioners exists, but little data supports safe productivity and supervision standards.*

What was the research question?

*What are the current productivity and supervision practices among emergency physicians, and are they perceived as safe?*

What was the major inding of the study?

*Most emergency physicians treat 2.6 patients per hour. Direct supervision increases perceived safety (OR 5.41, 95% CI: 1.27–25.55), while CMG employment decreases it (OR 0.24, 95% CI: 0.10–0.60).*

How does this improve population health?

*Findings guide safer supervision policies, ensuring patient care quality by aligning legislation with actual emergency physician workload capacities.*

For supervision models, direct supervision requires that the supervising physician see and evaluate every one of the supervisee’s patients over the course of their ED visit. Indirect supervision requires that the supervising physician discuss each patient with the supervisee during the course of clinical care, with or without personally evaluating the patient.^11^ Dependent variables were 1) EP assessment of the safety of patients per hour, reported as a binary (yes/no) variable, and 2) EP assessment of the safety of their workplace supervision model, also binary (yes/no). Supervision ratios were reported as attending EP:NPP or attending EP:resident physician.

### Data Analysis

Descriptive statistics were calculated and presented with summary information for the variables of interest and measures of spread. We estimated multivariate logistic regression models for factors associated with safety assessments both of productivity and supervision model. Finally, density plots were generated to compare reported safe staffing ratios by supervision model. We performed all analyses in R (R Foundation for Statistical Computing, Vienna, Austria).

## RESULTS

### Descriptive Statistics

A total of 196 EPs responded to our survey. Respondent characteristics are shown in Table 1. A plurality of respondents reported being fewer than 10 years out of residency, and most worked in EDs with mid-range annual patient visits. The overall mean patients per hour reported by EPs was 2.6, with a standard deviation of 1.7. Patients per hour were similar for physicians at different stages of their career, as well as across employment models, with the exception of hospital-employed community practice physicians, who saw 2.1 patients per hour. The self-reported patients per hour data was similar to recent data provided by the ED Benchmarking Alliance.^12^

On average, more than 80% of EPs felt that the number of patients they saw per hour was safe. This rate of safety assessment held for all subgroups except for community CMGs as an employer type and practice setting. Supervision models reported were direct, indirect, and retrospective chart review. Direct supervision represented 59% of total visits, and the majority of visits in all employment models with the exception of community CMGs. A minimum of 80% of physicians in all employment models felt that their supervision model was safe, again with the exception of community CMGs, in which fewer than two-thirds of physicians felt the supervision model was safe.

### Main Results

Table 2 shows the results for the multivariate logistic regression of determinants of safety assessment (yes/no) of the patients-per-hour staffing model. Overall, there were no significant associations between physician years out of residency or annual ED volume and assessment of safety of patients per hour. While the direction of the estimates for employment with a CMG and total patients per hour seen were in the expected direction, neither of these were significant at α=0.05. Table 3 shows the results for the multivariate logistic regression of determinants of safety assessment (yes/no) of the supervision model. As in the data for patients per hour, we found no association between years of practice and likelihood to report that supervision model was safe. However, a higher percentage of patients seen under direct supervision was associated with a higher likelihood of reporting a safe supervision model (odds ratio [OR] 5.41, 1.27–25.55). Furthermore, employment with a CMG was associated with less favorable assessments of the safety of supervision practices (OR 0.24, 0.10–0.60). Patients per hour was not statistically associated with assessment of supervision model safety. Figure shows side-by-side bar plots of the staffing ratios that EPs felt were safe. Physicians reported that they could safely supervise more emergency resident physicians than NPPs, with the most common safe resident ratios reported as 2:1 or 3:1. The most commonly reported safe staffing ratio for NPPs was 1:1 when directly supervised. A plurality (60/196) of emergency physicians reported that there was no safe staffing ratio for indirect supervision.

## DISCUSSION

Most survey respondents reported seeing an average of 2.6 patients per hour and practiced direct supervision with resident physicians and either direct or indirect supervision with NPPs. The majority of respondents felt that their productivity and supervision practices were safe. Those employed by community The CMGs were felt the least safe, comparatively, in terms of both productivity and supervision practices. Compared to other employment models, those who worked for CMGs practiced less direct supervision (27%).

To the best of our knowledge, this study is the only recent work reviewing EP productivity and supervision practices. Given the well-documented challenges with measuring safety and quality outcomes in EM,^10^,^13^ we used physicians’ own safety assessments of their practice model to evaluate trends. With respect to supervision ratios that EPs deemed to be safe, the response data approximated a normal distribution in which physicians felt that they could safely supervise 1–3 individuals at a time, with resident physicians considered to need less strict ratios than NPPs. Because our survey instrument did not include questions regarding the rationale for supervision ratios we were unable to determine with certainty the factors related to ratios considered safe. However, one hypothesis that might be considered in future work is that the level of training of the supervisee was a relevant factor in the safe ratio, with resident physicians having more formal training. Future work might also consider supervision ratios for newly graduated physician assistants and nurse practitioners relative to those with more experience.

Our findings raise two practical implications with respect to policymaking. First, it is essential that standards of care be established for the supervision of NPPs in the ED in the same manner that standards of care exist for the supervision of resident physicians. Supervision of resident physicians, as mandated by the Accreditation Council for Graduate Medical Education, must be a direct supervision model and adequate to provide individualized clinical instruction.^14^,^15^ Once standards are determined, legislation and hospital policies must be updated to reflect safe standards of care. No physician, no matter how talented, can supervise an unlimited number of other clinicians, and legislation permitting this practice places patients at risk. While our work is preliminary and limited in scope, early findings suggest that most EPs feel that they can safely supervise only 1–2 other clinicians at one time. Legislative changes that reflect these safety assessments must occur in parallel with further research on this topic.

The second policymaking implication is that standards of care for supervision must be properly incentivized, and liability for failure to meet standards must be properly assigned. Due to changes in the employment landscape in EM, emergency physicians themselves may have little control over productivity or supervision ratios. At the start of the specialty, most hospitals were owned in conjunction with medical schools, or by religiously affiliated nonprofit entities.^16^,^17^ Today, within the United States, nearly 5,000 hospitals have EDs. Most are managed by for-profit companies, and equity-backed CMGs staff many of those EDs.^16^ Of practicing EPs, just over 50% are employed while just under 30% have an ownership stake in their practice.^18^ In cases where substandard care results from unsafe productivity or supervision models, the unit of attribution for medico-legal purposes must be the party determining these models. In addition to the “stick” of liability, “carrots” of reimbursement tied to staffing ratios, similar to those in anesthesiology, might be considered. Anesthesiology has separate billing modifiers for services rendered by a physician vs a non-physician, in addition to modifiers for the number of procedures being supervised concurrently.^19^

## LIMITATIONS

Our study has several limitations. The first is that we had a limited number of survey respondents relative to total number of practicing EPs, and our sampling method did not have the capability to randomly select among practicing physicians. We elected to limit our sampling frame to AAEM membership to limit non-randomness of the sample, but this decision naturally limited the potential number of respondents. Future work should expand on sample size. A second limitation is that there is a great deal of variability both in staffing models and terminology regarding supervision, so some physicians may have interpreted terms such as indirect or direct in ways other than intended despite provided definitions.

More generally, while our survey was developed iteratively with feedback from stakeholders, the instrument has not been validated. We did not have a survey item related to the level of experience of NPPs or the training level of resident physicians and, thus, were unable to determine whether the experience and training of supervisees was a factor in safety assessments. Finally, no respondents reported being resident physicians. To the extent that some residents may supervise junior residents or students, our data may not represent the views of this population. Lack of participation from resident physicians may be a result of our sampling strategy, a perception that the survey was directed more toward faculty physicians, or a lack of empowerment to raise concerns about NPP staffing, as noted in recent work.^20^

## CONCLUSION

Our survey found that, on average, most emergency physicians treated 2.6 patients per hour and that productivity was similar across years of clinical experience, employment model, and supervision model. Most EPs felt that their productivity and supervision practices were safe, although EPs employed by community contract-management groups felt less safe with regard to both productivity and supervision compared to those in other employment models. Our assessment of EP productivity and supervision practices should be considered when developing future guidelines, policies, and legislation that impact emergency care. Furthermore, clear standards of care with respect to supervision must be established and could be linked to reimbursement in the future.

## Supplementary Information





## Figures and Tables

**Figure:**
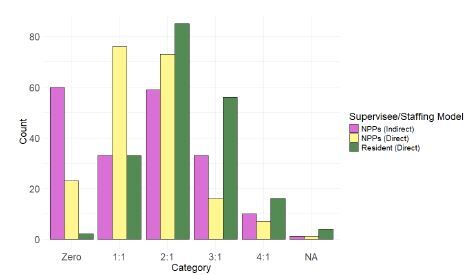
Safe supervision ratios for resident physicians and non-physician practitioners. NPP, non-physician practitioner.

**Table 1:** Characteristics of survey respondents and descriptive staffing variables.

Characteristics of survey respondents		Staffing and safety variables			
	
Characteristic	No. (%)	Patients per hour (SD)	Patients per hour safety assessment (%)	Supervision model % direct (SD)	Supervision model safety assessment (%)
Years out of residency					
Still in residency	0 (0%)	NA	NA	NA	NA
0–10	77 (39%)	2.5 (1.2)	63/77 (82%)	58 (33.1)	60/77 (78%)
11–20	50 (26%)	2.6 (1.0)	43/50 (86%)	63 (36.5)	43/50 (86%)
21–30	45 (23%)	2.6 (2.5)	35/45 (78%)	65 (31.9)	40/45 (89%)
>31	24 (12%)	3.2 (2.6)	22/24 (92%)	50 (33.1)	21/24 (88%)
Annual volume at primary practice setting					
<20,000	24 (12%)	1.5 (0.6)	23/24 (96%)	71 (41.8)	23/24 (96%)
20,001–50,000	54 (28%)	2.4 (0.8)	42/54 (78%)	49 (30.8)	39/54 (72%)
50,001–75,000	54 (28%)	3.2 (2.7)	47/54 (87%)	53 (33.3)	46/54 (85%)
75,001–100,000	45 (23%)	2.9 (1.3)	34/45 (76%)	70 (30.8)	40/45 (89%)
>100,000	19 (10%)	2.5 (1.1)	17/19 (89%)	71 (32.2)	16/19 (84%)
Employment type					
Hospital employed					
Academic	38 (19.4%)	2.9 (1.3)	31/38 (82%)	70 (29.4)	33/38 (87%)
Community	50 (25.5%)	2.1 (1.2)	43/50 (86%)	51 (35.4)	44/50 (88%)
Contract Management Group (Envision, Team Health, etc.)					
Academic	6 (3.1%)	2.8 (0.9	5/6 (83%)	71 (24.1)	6/6 (100%)
Community	40 (20.4%)	2.7 (1.8)	30/40 (75%)	45 (29.8)	25/40 (63%)
Democratic group					
Academic	5 (2.6%)	2.8 (0.9)	4/5 (80%)	87 (28.1)	5/5 (100%)
Community	52 (26.5%)	2.8 (2.4)	46/52 (88%)	64 (34.0)	47/52 (90%)
Military	5 (2.6%)	3.0 (1.0)	4/5 (80%)	67 (47.0)	4/5 (80%)
Total	196 (100%)	2.6 (1.7)	163/196 (83%)	59 (33.8)	164/196 (84%)

**Table 2:** Multivariate logistic regression results: safety of patients per hour.

Variable	Estimate (95% CI)	Odds ratio (95% CI)
Characteristics of survey respondent		
Years out of residency		
	0.00 [−0.03, 0.04]	1.00 [0.97, 1.04]
Annual ED volume (reference: 20,001–50,000 patient visits)		
<than 20,000	1.33 [−0.49, 4.30]	3.77 [0.61, 73.73]
50,001–75,000	0.86 [−0.22, 2.03]	2.36 [0.81, 7.58]
75,001–100,000	−0.21 [−1.27, 0.85]	0.81 [0.28, 2.34]
>100,000	1.49 [−0.32, 4.45]	4.42 [0.73, 85.45]
Employment type (reference: non-contract management group)		
Contract Management Group	−0.91 [−1.83, 0.02]	0.40 [0.16, 1.02]
Staffing and productivity		
Total patients per hour	−0.12 [−0.33, 0.10]	0.88 [0.72, 1.11]
Supervision		
% patients seen primarily by a physician or under direct supervision in workplace	0.41 [−0.93, 1.77]	1.51 [0.39, 5.86]

*CI*, confidence interval; *ED*, emergency department.

**Table 3:** Multivariate logistic regression results: safety of supervision model.

Variable	Estimate (95% CI)	Odds ratio (95% CI)
Characteristics of survey respondent		
Years out of residency		
	0.03 [−0.01, 0.07]	1.03 [0.47, 5.21]
Annual ED volume (reference: 20,001–50,000 patient visits)		
<than 20,000	1.45 [−0.38, 4.42]	4.26 [0.68, 83.00]
50,001–75,000	1.05 [−0.02, 2.22]	2.87 [0.98, 9.18]
75,001–100,000	0.63 [−0.57, 1.94]	1.88 [0.56, 6.96]
>100,000	0.76 [−0.84, 2.78]	2.13 [0.43, 16.13]
Employment type (reference: non-contract management group)		
Contract Management Group	−1.42 [−2.35, −0.50]*	0.24 [0.10, 0.60]**
Staffing and productivity		
Total patients per hour	−0.12 [−0.33, 0.14]	0.89 [0.72, 1.16]
Supervision		
% patients seen primarily by a physician or under direct supervision in workplace	1.69 [0.24, 3.24]*	5.41 [1.27, 25.55]**

* 95% confidence interval for estimate does not cross zero.

** 95% confidence interval for odds ratio does not cross one.

*CI*, confidence interval**;** *ED*, emergency department.
