# Supplementary material for: Emergency Physician Assessment of Productivity and Supervision Practices
Source: West J Emerg Med. 2025 Apr 1;26(3):500–6. doi: 10.5811/westjem.19417 (PMC12208038; doi:10.5811/westjem.19417)
Supplement: Supplementary file 1 [file wjem-26-500-s001.docx]

**Appendix 1 – Survey**

1. Name and Email Address
2. Gender
   1. Female
   2. Male
   3. Non-binary
   4. Prefer not to disclose
   5. Prefer to self-describe
   6. Unknown
3. Race
   1. American Indian or Alaska Native
   2. Asian
   3. Black or African American
   4. Native Hawaiian or Other Pacific Islander
   5. White
   6. Unknown
   7. Other:
4. Ethnicity
   1. Hispanic or Latino/x
   2. Not Hispanic or Latino/x
   3. Unknown
   4. Other:
5. Years out of residency
   1. 0-65
6. Employment model for your primary workplace
   1. CMG (Envision, Team Health, SCP, etc.)
   2. Democratic Group
   3. Military
   4. Hospital Employed
7. Primary setting for your primary workplace
   1. Community
   2. Academic
8. Annual patient volume in your primary workplace
   1. Less than 20,000
   2. 20,001-50,000
   3. 50,001-75,000
   4. 75,001-100,000
   5. Greater than 100,000
9. **Your** **current** **total** patients per hour (primary patients seen by an attending physician without a supervisee plus directly supervised patients, evaluated independently by the supervisor in addition to the supervisee)What is the maximum number of residents you supervise at one time?
10. **Your current average*primary*** patients per hour (primary patients are those seen by an attending physician without a supervisee) Describe your supervision model of NPs/PAs (i.e. Direct supervision, indirect supervision/discussing cases in real time, retrospective chart review, etc.)
11. **Your current average *directly supervised*** patients per hour (directly supervised patients are those evaluated independently by the supervisor, in addition to the supervisee) Would you consider your current practice of supervision safe? If not, what would you change?
12. On average in **your primary workplace**, how many patients per hour do you estimate have ***direct attending physician supervision***?  (directly supervised patients are those evaluated independently by the supervisor, in addition to the supervisee) What do you feel is a safe NPP to physician supervision ratio for indirect supervision of patients (where the physicians do not necessarily see every patient)?
13. On average in**your primary workplace,** how many patients per hour do you estimate have ***indirect attending physician supervision***?  (indirect supervision requires that the supervisor discusses each patient with the supervisee, but may or may not personally evaluate the patient) During one shift, how many total presenters undergoing direct supervision do you think is safe, including NP/PA/residents?
14. On average in **your primary workplace**, how many patients per hour do you estimate do **not**have any real-time attending physician supervision? (those who undergo, at most, retrospective chart review and are not directly or indirectly supervised)
15. Would you consider your current practice of patients per hour safe?
    1. Yes
    2. No
16. Would you consider your current practice of supervision safe?
    1. Yes
    2. No
17. What do you feel is a safe NPP to attending physician supervision ratio for direct supervision of patients? (directly supervised patients are those evaluated independently by the supervisor, in addition to the supervisee)
    1. Zero (o)
    2. 1:1
    3. 2:1
    4. 3:1
    5. 4:1
    6. Greater than 4:1
18. What do you feel is a safe NPP to attending physician supervision ratio for indirect supervision of patients?  (indirect supervision requires that the supervisor discusses each patient with the supervisee, but may or may not personally evaluate the patient)
    1. Zero (o)
    2. 1:1
    3. 2:1
    4. 3:1
    5. 4:1
    6. Greater than 4:1
19. What do you feel is a safe resident physician to attending physician supervision ratio?
    1. Zero (o)
    2. 1:1
    3. 2:1
    4. 3:1
    5. 4:1
    6. Greater than 4:1
